# Cross-reactive human antibody responses to H9N2 influenza virus, New York, United States, 2025

**DOI:** 10.2807/1560-7917.ES.2026.31.20.2600375

**Published:** 2026-05-21

**Authors:** Gagandeep Singh, Disha Bhavsar, Lucas M Ferreri, Jessica R Nardulli, Charles Gleason, Neko Lyttle, Yuexing Chen, Anice C Lowen, Viviana Simon, Florian Krammer

**Affiliations:** 1 Department of Microbiology, Icahn School of Medicine at Mount Sinai (ISMMS), New York, United States; 2 Center for Vaccine Research and Pandemic Preparedness (C-VaRPP), Icahn School of Medicine at Mount Sinai (ISMMS), New York, United States; 3 Department of Microbiology and Immunology, Emory University School of Medicine, Atlanta, United States; 4 Division of Infectious Diseases, Department of Medicine, Icahn School of Medicine at Mount Sinai (ISMMS), New York, United States; 5 The Global Health and Emerging Pathogens Institute, Icahn School of Medicine at Mount Sinai (ISMMS), New York, United States; 6 Department of Pathology, Molecular and Cell-Based Medicine, Icahn School of Medicine at Mount Sinai, New York, United States; 7 Ignaz Semmelweis Institute, Interuniversity Institute for Infection Research, Medical University of Vienna, Vienna, Austria; 8 Ludwig Boltzmann Institute for Science Outreach and Pandemic Preparedness at the Medical University of Vienna, Vienna, Austria

**Keywords:** H9N2, zoonoses, avian influenza, influenza A virus

## Abstract

Understanding population immunity to emerging viruses is critical for risk assessment. We characterised antibody responses to avian influenza A(H9N2) viruses in 298 human sera from adults in New York City. We observed widespread cross-reactive H9-binding antibodies, albeit at lower levels (GMT = 371.3) than against seasonal H3 (GMT = 1,398.0), with low to undetectable haemagglutination inhibition and neutralising antibodies. In contrast, NA-binding and neuraminidase inhibition responses were moderate (GMT = 149.5 and 34.2) and comparable to those against human seasonal N2 (GMT = 259.0 and 42.2).

Avian influenza A(H9N2) viruses have been endemic in poultry for many years across Asia, the Middle East, and Northern and Western Africa and have been detected periodically in the Americas and Europe. Since their first detection in 1966, H9N2 viruses have evolved into multiple lineages and exhibit a high propensity for reassortment, contributing internal genes to zoonotic viruses such as H5N1, H7N9 and H10N8 [1,2]. Human infections with H9N2 viruses have been reported since 1998, with most cases linked to poultry exposure and typically resulting in mild or asymptomatic disease but severe cases and deaths have been observed as well. Despite these observations, population-level immunity to H9N2 remains poorly defined, particularly in regions without endemic exposure to the virus. Here, we assessed cross-reactive antibody responses to H9N2 viruses using a panel of recently collected human sera from the general population of a large metropolitan city in North America (New York City).

## Collection and analysis of sera

We collected sera between February 2024 and April 2025 from adult participants living or working in New York City as part of the APOLLO (Antibody panels of longitudinal levels of coronavirus immunity) observational study conducted at the Icahn School of Medicine at Mount Sinai. All participants provided written informed consent before sample and data collection. In total, 298 adults born between 1938 and 2005 were included (214 females, 84 males). Vaccination history was not considered in the biospecimen selection. To assess cross-reactive haemagglutinin (HA)- and neuraminidase (NA)-specific antibody responses to a lineage B4.7.2 H9N2 virus (A/Changsha/SR353/2025), sera were analysed by ELISA, HA inhibition (HI), microneutralisation (MNT) and NA inhibition (NI) assays [3]. For comparison, we also determined HA- and NA-specific antibody responses to the 2024/25 influenza vaccine strain (Northern hemisphere) A/Thailand/8/2022 (H3N2), with NI performed using a reassortant H6N2 (A/Singapore/INFIMH/16–0019/2016) virus. All statistical analyses, area under the curve (AUC) calculation, and median inhibitory dilution (ID_50_) calculations were conducted with Graphpad Prism version 10 (https://www.graphpad.com/updates/prism-1000-release-notes). Differences between antibody titres between three groups were analysed with a Kruskal–Wallis test with Dunn’s multiple comparisons.

## Binding antibody responses to H9 and N2

**Figure 1:**
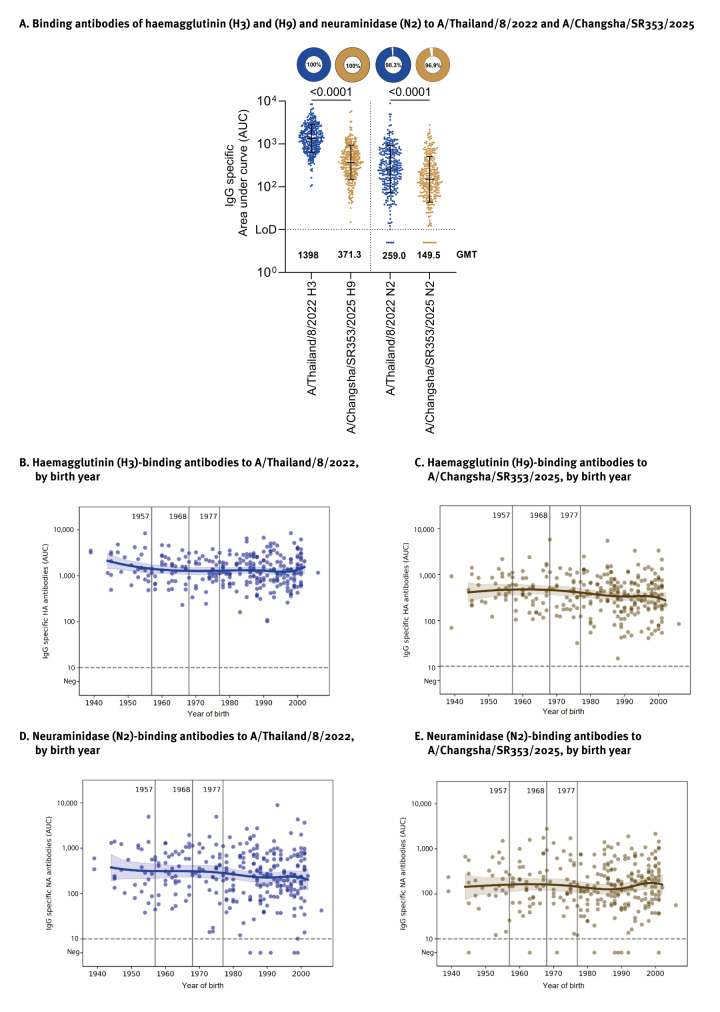
Influenza A virus haemagglutinin (H3, H9)- and neuraminidase (N2)-specific binding antibody responses in human sera, New York City, United States, February 2024–April 2025 (n = 298)

AUC: area under the curve; GMT: geometric mean titre; GSD: geometric standard deviation; HA: haemagglutinin; LOD: limit of detection; NA: neuraminidase.

First, we measured binding antibodies to lineage B4.7.2 H9 HA and N2 NA in all 298 human sera using an ELISA. The H9 and N2 antigens used were derived from A/Changsha/SR353/2025, a strain isolated from a human infection in 2025. As expected, reactivity to H3 HA was higher (Figure 1A; geometric mean titre (GMT) = 1,398.0) whereas reactivity to H9 HA was significantly lower (GMT = 371.3; p < 0.0001 vs H3N2). Similarly, reactivity to N2 from A/Changsha/SR353/2025 (H9N2) was lower (GMT = 149.5; p < 0.0001 vs H3N2) than that of A/Thailand/8/2022 N2 (GMT = 259.0). These findings substantiate that the study participants had pre-existing HA and NA antibodies likely elicited by seasonal influenza virus infection and vaccination which are cross-reactive to zoonotic influenza subtypes [3-6]. When we stratified titres by age, we did not observe major apparent birth year-related differences in antibody reactivity to either antigen (Figure 1B-E). This likely reflects widespread and repeated exposure to H3N2 viruses across all age groups, resulting in broadly distributed immunity. However, H9N2 responses are likely driven by cross-reactive antibodies rather than direct exposure, leading to a more uniform distribution across birth years. Smaller effects could also have been masked by the relatively low number of individuals in the study.

Panel A. IgG binding antibody responses were measured by ELISA using recombinant HA and NA proteins from influenza A/Thailand/8/2022 (H3N2) and lineage B4.7.2 A/Changsha/SR353/2025 (H9N2) viruses. Antibody levels are presented as AUC, with GMT values and GSD indicated by error bars. Dashed lines denote assay LOD. The proportion of samples exceeding the LOD for each antigen is summarised in pie charts, with percentages shown within each chart. AUC values were log10-transformed before analysis. Differences in binding responses across antigens were assessed using the Kruskal–Wallis test followed by Dunn’s multiple-comparison correction; p values are indicated.

Panels B–E. Birth year stratified HA- and NA-binding antibody responses. Vertical reference lines indicate the emergence of influenza A H2N2 (1957), H3N2 (1968), and the re-emergence of H1N1 viruses (1977). Each point represents an individual serum sample, and smoothed trends are shown using locally estimated scatterplot smoothing (LOESS).

## Haemagglutination inhibition and microneutralisation titres to lineage B4.7.2 H9N2 virus

**Figure 2:**
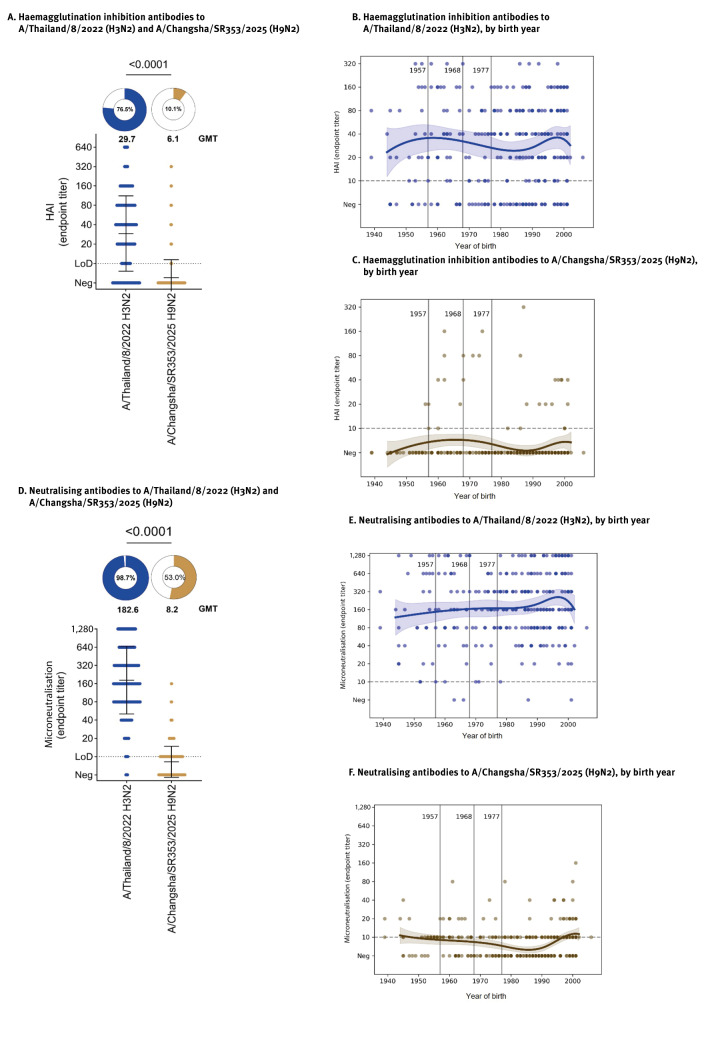
Haemagglutination inhibiting and neutralising antibody responses against influenza A (H3N2) and (H9N2) viruses in human sera, New York City, United States, February 2024–April 2025 (n = 298)

GMT: geometric mean titre; GSD: geometric standard deviation; HA: haemagglutinin; HI: haemagglutination inhibition; LOD: limit of detection.

We next measured HI antibodies, which block virus attachment to host cells and represent an established correlate of protection [7]. In addition, we performed in vitro microneutralisation assays to assess antibody-mediated inhibition of viral replication through additional mechanisms e.g. contributions from anti-NA and anti-stalk specific HA antibodies. As expected, 228 (76.5%) individuals had HI titres against the H3N2 virus (GMT = 29.7), whereas only 30 (10.1%) had detectable HI titres against H9N2 lineage B4.7.2 A/Changsha/SR353/2025 (GMT = 6.1) (Figure 2A). Similarly, 294 (98.7%) sera showed detectable neutralising antibody titres against H3N2 (GMT = 182.6), whereas only 158 (53.0%) had detectable neutralising titres against A/Changsha/SR353/2025 H9N2 (GMT = 8.2) with most samples at or below the limit of detection (Figure 2D). We next stratified the HI and microneutralisation data by birth year: interestingly, there was a modest increase in HI and MNT titres for both viruses in individuals born after 1990 (105 individuals) (Figure 2B-C, E-F).

Panel A. HI antibody responses of human sera against influenza A/Thailand/8/2022 (H3N2) and A/Changsha/SR353/2025 (H9N2) viruses. HI titres are presented as endpoint titres, with GMTs shown and error bars indicating GSD. The assay LOD was 1:10; samples below the LOD were assigned a value of 5 for GMT calculation. LODs are indicated by dotted lines. The proportion of serum samples with HI titres above the LOD is shown in pie charts, with percentages displayed within the charts. Statistical significance between viruses was determined using the Kruskal–Wallis test followed by Dunn’s multiple-comparison correction; p values are indicated above brackets.

Panel D. Multicycle microneutralisation assays were performed to assess antibody responses against A/Thailand/8/2022 (H3N2) and A/Changsha/SR353/2025 (H9N2) influenza viruses. Titres are presented as endpoint titres, with GMTs indicated. LODs are shown as dotted lines. The proportion of samples with titres above the LOD is summarised in pie charts, with percentages displayed below each chart. Statistical significance between viruses was determined using the Kruskal–Wallis test followed by Dunn’s multiple-comparison correction; p values are indicated above brackets.

Panels B–C, E–F. HI and microneutralisation antibody titres stratified by year of birth. Vertical lines indicate the emergence of influenza A virus H2N2 (1957), H3N2 (1968) and re-emergence of H1N1 (1977) influenza viruses. Each point represents an individual serum sample, and locally estimated scatterplot smoothing (LOESS) curves are shown.

## Neuraminidase inhibition titres to H9N2 virus

**Figure 3:**
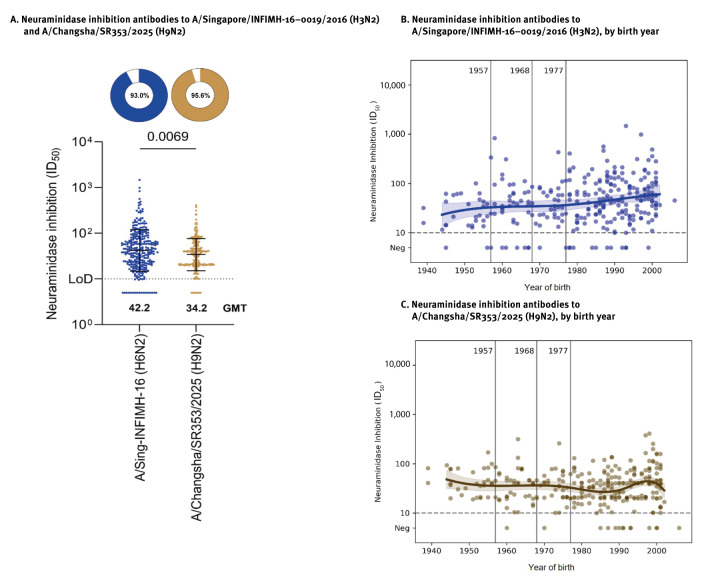
Functional neuraminidase inhibition antibody responses to N2 of influenza A(H3N2) and lineage B4.7.2 (H9N2) viruses in human sera, New York City, United States (n = 298)

GMT: geometric mean titre; HA: haemagglutinin; ID_50_: median inhibitory dilution; LOD: limit of detection; NI: neuraminidase inhibition.

Antibodies targeting NA typically inhibit the neuraminidase enzymatic activity [8]; therefore, we performed enzyme-linked lectin assays (ELLAs) to measure antibodies that inhibit NA-mediated cleavage of sialic acids on fetuin [9]. Neuraminidase inhibition (NI) antibodies against H9N2 were detected in 285 (95.6%) serum samples (GMT = 34.2). To assess pre-existing population-level seasonal H3N2 virus NA immunity without interference from anti-HA antibodies, we used a reassortant H6N2 (A/Singapore/INFIMH/16–0019/2016) virus, as H6 HAs are not commonly encountered in humans to minimise confounding by HA-directed immunity [10]. Antibodies (NI) against H6N2 were detected in 277 (93.0%) serum samples (GMT = 42.2) (Figure 3A). When stratified by birth year, titres were highest among the youngest individuals (Figure 3B-C).

Panel A. NI antibody responses were measured by enzyme-linked lectin assay (ELLA) using a reassortant virus expressing mismatched HA and target N2 neuraminidase from A/Singapore/INFIMH-16–0019/2016 (H3N2) and the lineage B4.7.2 A/Changsha/SR353/2025 (H9N2) virus. NI titres are presented as ID_50_ values, with GMTs indicated. LODs are shown as dotted lines. The proportion of serum samples with ID_50_ values above the LOD is summarised in pie charts, with percentages displayed below each chart. NI titres across viruses were compared using the Kruskal–Wallis test followed by Dunn’s multiple-comparison correction; adjusted p values are indicated above brackets.

Panels B–C. NI titres stratified by year of birth. Vertical reference lines indicate the emergence of H2N2 (1957), H3N2 (1968), and the re-emergence of H1N1 (1977). Each point represents an individual serum sample, and smoothed trends are shown using locally estimated scatterplot smoothing (LOESS).

## Discussion

A key parameter of risk assessment for avian influenza viruses in humans is an in-depth comprehensive understanding of pre-existing immunity at the population level. Natural infection with influenza viruses in humans has been shown to induce immune responses of greater magnitude, breadth, and durability than influenza vaccination [11]. Cross-reactive and cross-protective antibodies have become the focus of influenza virus research, as they can inform the development of broadly protective vaccines and therapeutics [6]. Here, we demonstrate how seasonal influenza virus infection and vaccination shape the cross-reactive antibody repertoire.

Zoonotic H9N2 infections in humans have been reported since the late 1990s, are usually linked to exposure to infected birds and typically lead to mild disease. However, severe illness and rare fatalities have been reported, particularly in individuals with underlying conditions [12]. Serological studies indicate that human exposure may be more widespread than indicated by confirmed infections [13,14]. In April 2025, three laboratory-confirmed human H9N2 cases were identified in Changsha, China. All cases were sporadic and mild, with no evidence of sustained human-to-human transmission. Genetic analysis showed that the viruses belonged to the Y280-like lineage and harboured mutations associated with increased binding to human-type α2,6-linked sialic acid receptors, highlighting their zoonotic potential [15]. In addition, a recent report of an imported human H9N2 case in Italy in March 2026, identified in a patient with a weakened immune system, underscores the expanding geographic footprint of these viruses and the continued risk of cross-border transmission [16].

Our work describes pre-existing immunity at population level to avian influenza A(H9N2) viruses in adults from New York City. We observed widespread cross-reactive haemagglutinin (HA)-binding antibodies in the sampled group; however, these responses were of lower magnitude than those against seasonal H3. Consistent with this, HI and neutralising antibody responses to H9N2 were low or undetectable in most individuals, indicating limited functional immunity.

In contrast, a high proportion of individuals exhibited N2-binding and NI antibodies, including cross-reactive responses to H9N2. Although NA-directed antibodies do not prevent infection, they may reduce viral replication and disease severity, potentially providing the population with some degree of resilience against emerging influenza viruses [6,7]. The comparable magnitude of NI responses to H9N2 and H3N2 suggests that prior exposure to seasonal influenza viruses has generated cross-reactive NA immunity. This is consistent with historical observations that pre-existing anti-N2 antibodies, induced during circulation of A(H2N2) viruses, may have contributed to reduced disease severity during the emergence of the A(H3N2) pandemic [17,18].

The difference between HA and NA responses is not surprising. Comparative sequence analysis revealed substantial divergence between the HA proteins of lineage B4.7.2 virus A/Changsha/SR353/2025 (H9N2) and seasonal A/Thailand/8/2022 (H3N2), with only approximately 40% amino acid identity. In contrast, the N2 neuraminidases of the two viruses showed high conservation, sharing approximately 89% amino-acid identity.

Our study also has several limitations. Vaccination data were not captured, and we therefore cannot draw any conclusions about the effect of seasonal vaccination on H9N2 immunity. Furthermore, our analysis was restricted to a small, geographically limited group of individuals. Finally, different lineages of H9N2 circulate and they are antigenically distinct. This study only used one of these viruses as reagent and cross-reactive responses to other lineages of H9N2 may differ.

## Conclusion

In summary, we found that pre-existing immunity to H9N2 in humans that is dominated by cross-reactive but largely non-neutralising antibodies, with a greater contribution from NA-directed responses. Given the pandemic potential of H9N2 viruses, these findings have important implications for risk assessment and support the inclusion of NA immunity in vaccine development strategies. Further studies are needed to confirm whether cross-reactive NI antibodies confer protection against H9N2 infection or modulate disease severity, but our results suggest that the antibodies against H9N2 virus might derive from exposure to conserved epitopes shared between the avian-origin and seasonal strains.

## Data Availability

All data described in this article can be found on Zenodo under identifier https://doi.org/10.5281/zenodo.20310846.
